# Qiangji Jianli Decoction Alleviates Hydrogen Peroxide-Induced Mitochondrial Dysfunction via Regulating Mitochondrial Dynamics and Biogenesis in L6 Myoblasts

**DOI:** 10.1155/2021/6660616

**Published:** 2021-04-13

**Authors:** Jingwei Song, Qing Li, Lingling Ke, Jian Liang, Wei Jiao, Huafeng Pan, Yanwu Li, Qun Du, Yafang Song, Aidong Ji, Zhiwei Chen, Jinqiu Li, Lanqi Li

**Affiliations:** ^1^Institute of Pi-Wei, Science and Technology Innovation Center, Guangzhou University of Chinese Medicine, Guangzhou 510405, China; ^2^Guangdong Provincial Key Laboratory of New Drug Development and Research of Chinese Medicine, Mathematical Engineering Academy of Chinese Medicine, Guangzhou University of Chinese Medicine, Guangzhou, 510006 Guangdong, China; ^3^Clinical Medical College of Acupuncture, Moxibustion and Rehabilitation, Guangzhou University of Chinese Medicine, Guangzhou 510006, China

## Abstract

Oxidative stress can cause the excessive generation of reactive oxygen species (ROS) and has various adverse effects on muscular mitochondria. Qiangji Jianli decoction (QJJLD) is an effective traditional Chinese medicine (TCM) that is widely applied to improve muscle weakness, and it has active constituents that prevent mitochondrial dysfunction. To investigate the protective mechanism of QJJLD against hydrogen peroxide- (H_2_O_2_-) mediated mitochondrial dysfunction in L6 myoblasts. Cell viability was determined with MTT assay. Mitochondrial ultrastructure was detected by transmission electron microscope (TEM). ROS and mitochondrial membrane potential (MMP) were analyzed by fluorescence microscope and flow cytometry. The superoxide dismutase (SOD), glutathione peroxidase (GSH-Px) activity, and malondialdehyde (MDA) level were determined by WST-1, TBA, and DTNB methods, respectively. The mRNA and protein levels were measured by quantitative real-time PCR (qRT-PCR) and Western blot. The cell viability was decreased, and the cellular ROS level was increased when L6 myoblasts were exposed to H_2_O_2_. After treatment with QJJLD-containing serum, the SOD and GSH-Px activities were increased. MDA level was decreased concurrently. ROS level was decreased while respiratory chain complex activity and ATP content were increased in L6 myoblasts. MMP loss was attenuated. Mitochondrial ultrastructure was also improved. Simultaneously, the protein expressions of p-AMPK, PGC-1*α*, NRF1, and TFAM were upregulated. The mRNA and protein expressions of Mfn1/2 and Opa1 were also upregulated while Drp1 and Fis1 were downregulated. These results suggest that QJJLD may alleviate mitochondrial dysfunction through the regulation of mitochondrial dynamics and biogenesis, the inhibition of ROS generation, and the promotion of mitochondrial energy metabolism.

## 1. Introduction

Mitochondria have great impact on longevity and stress resistance [1]. The mitochondrial membrane potential (MMP) and the activity of mitochondrial respiratory chain complexes are crucial for maintenance of mitochondrial function. Mitochondria provide main energy supply in muscle cells. Yu and Yang have demonstrated that AMPK played a key role in mitochondrial energy metabolism by modulating PGC-1*α* [2]. They found PGC-1*α* acted as a master transcription factor for mitochondrial biogenesis by increasing the expression of NRF1 and TFAM, and PGC-1*α* is also important for the expression of nuclear genes that encode respiratory chain subunits. Both mitochondrial biogenesis and the activities of the mitochondrial respiratory chain are important for the increase in intracellular ATP levels.

Mitochondria are present in the mammalian cells and display a continuous cycle of fission and fusion, which are known as mitochondrial dynamics. Fusion is controlled by mitochondrial fusion protein mitofusin-1 (Mnf1), mitochondrial fusion protein mitofusin-2 (Mfn2), and optic atrophy 1 (Opa1). Dynamin-related protein 1 (Drp1) and fission protein 1 (Fis1) are the best-known proteins that regulate mitochondrial fission. The mitochondrial network changes mitochondrial shape through fusion and fission to accommodate metabolic requirements [3]. The balance of these two events provides an equilibrium of mitochondrial networks and is thought to be essential for mitochondrial homeostasis, cell stability, and cell survival. Changes in mitochondrial dynamics-related proteins will alter mitochondrial morphology. For example, downregulation of Opa1 with siRNA may lead to cristae breakage, resulting in mitochondrial fragmentation [4]. Decreased Drp1 and Fis1 levels induced a mitochondrial elongation and protected mitochondria from oxidative stress [5]. Changes in mitochondrial dynamics-related proteins may also affect the mitochondrial energy metabolism. Abnormal expression of Mfn2 may impair energy metabolism by reducing the transport speed of mitochondria in motor axons and sensory axons [6]. Opa1 mutation induces a coupling defect of oxidative phosphorylation (OXPHOS) and a faint decrease in ATP production [7].

Qiangji Jianli decoction (QJJLD) has been used clinically for more than 30 years and consists of Astragalus mongholicus Bunge, Codonopsis pilosula (Franch.) Nannf., Atractylodes macrocephala Koidz., Angelica sinensis (Oliv.) Diels, Actaea cimicifuga L., Bupleurum chinense DC., Citrus auranthium L., and Glycyrrhiza uralensis Fisch [8]. It is reported that QJJL capsules can attenuate various types of muscle injury, such as muscle fiber rupture, denaturalization, muscle necrosis, and atrophy [9]. In our previous study, we found some studies reported that Astragalus mongholicus Bunge, Bupleurum chinese DC, and Glycyrrhiza uralensis Fisch have protective effects for mitochondria, like attenuating the MPP loss, inhibiting ROS generation, or maintaining mitochondrial energy metabolism [10]. Our previous experimental results also indicated that QJJLD can improve the ultrastructure of skeletal muscle fibers and mitochondria in rats with myasthenia gravis. The enzymatic activity of mitochondrial respiratory chain complexes I-IV was also improved which indicates that QJJLD could regulate mitochondrial energy metabolism.

However, can QJJLD act on the skeletal muscle cells to against mitochondrial dysfunction? What is the mechanism by which it regulates mitochondrial energy metabolism? The answers are still unknown. Investigating these crucial questions may be helpful to provide a new therapy for myopathy. Therefore, the current study was designed to determine the protective effect of QJJLD on skeletal muscle cells. To make mitochondria dysfunction, rat L6 myoblasts were exposed to hydrogen peroxide for 1 h. The MTT colorimetric analysis was applied to detect the cell viability. Then, MMP, the enzymatic activity of mitochondrial respiratory chain complexes I-IV, ROS level, and ATP content in L6 myoblasts were used to estimate mitochondrial function. mRNA and protein expression levels of Mfn1/2, Opa1, Drp1, and Fis1 were examined through PCR and Western blot analyses, respectively. The protein expression levels of p-AMPK, COX IV, PGC-1*α*, NRF1, and TFAM were also detected by Western blot analysis. Our study planned to investigate whether QJJLD regulates mitochondrial dynamics and mitochondrial biogenesis to repair mitochondrial dysfunction and thus to improve mitochondrial energy metabolism in muscle cell.

## 2. Materials and Methods

### 2.1. Drugs and Reagents

All Chinese herbs were bought from Guangzhou Xingyuanchun Pharmacy (Guangzhou, China) and passed the quality appraisal of the College of Chinese Pharmaceutical Science (Guangzhou University of Chinese Medicine). QJJLD consists of eight herbs: Astragalus mongholicus Bunge (voucher number: 18-10-3), Angelica sinensis (Oliv.) Diels (voucher number: 18-10-4), Codonopsis pilosula (Franch.) Nannf. (voucher number: 18-10-5), Bupleurum chinense DC. (voucher number: 18-10-6), Glycyrrhiza uralensis Fisch (voucher number: 18-10-7), Atractylodes macrocephala Koidz. (voucher number: 18-10-8), Actaea cimicifuga L. (voucher number: 18-10-9), and Citrus auranthium L. (voucher number: 18-10-10). The standards calycosin-7-glucoside (CAS: 20633-67-4), liquiritin (CAS: 551-15-5), ferulic acid (CAS: 1135-24-6), isoferulic acid (CAS: 537-73-5), hesperidin (CAS: 520-26-3), lobetyolin (CAS: 136085-37-5), astragaloside IV (CAS: 84687-43-4), monoammonium glycyrrhizinate (CSA: 53956-04-0), saikosaponin A (CAS: 20736-09-8), saikosaponin D (CAS: 20874-52-6), and atractylenolide III (CAS: 73030-71-4) were purchased from Nanjing Yuanzhi Biotechnology Co., Ltd (Nanjing, China). DMEM (CAS: C11995500BT) was purchased from Gibco (New York, USA). FBS (CAS: 10099-141) was purchased from HyClone (Utah, USA). MTT (CAS: 298-93-1) and DMSO (CAS: 67-68-5) were purchased from Amresco (OH, USA). ROS assay kit (CAS: E004-1-1), SOD assay kit (CAS: A001-3-2), GSH-Px assay kit (CAS: A005-1-2), MDA assay kit (CAS: A003-1-2), mitochondrial respiratory chain complexes I-IV assay kit (complex I: CASA089-1-1, complex II: CASA089-2-1, complex III: CASA089-3-1, and complex IV: CASA089-4-1), and ATP assay kit (CAS: A095-1-1) were purchased from Nanjing Jiancheng Bioengineering Institute (Nanjing, China). JC-1 Mitochondrial membrane potential assay kit (CAS: BB-4105-50T) was obtained from Bestbio Biotechnology Co., Ltd (Shanghai, China). Epon 812 resin (CAS: GP18010) was purchased from Zhongjingkeyi Technology Co., Ltd (Beijing, China). TRNzol-A+ reagent (CAS: DP421) was purchased from TIANGEN Biotechnology Co., Ltd (Beijing, China). PrimeScript™ RT Master Mix Kit (CAS: RR036A) and SYBR Premix Ex Taq™ II (CAS: RR820A) were purchased from TaKaRa Biotechnology Co., Ltd (Tokyo, Japan). BCA Protein Assay Kit (CAS: KGP902) was purchased from KeyGEN (Jiangsu, China). Chemiluminescence solution (CAS: P0018A, ECL) was purchased from Beyotime Biotechnology Co., Ltd (Shanghai, China). Primary antibodies AMPK (CAS: ab80039), p-AMPK (CAS: ab133448), PGC-1*α* (CAS: ab54481), NRF1 (CAS: ab175932), TFAM (CAS: ab131607), and GAPDH (CAS: ab8245) were purchased from Abcam Biotechnology. Antibody against COX IV (CAS: 4850) was purchased from Cell Signaling Technology, Inc. (California, USA). Antibodies against Mfn1 (CAS: sc-166644), Mfn2 (CAS: sc-515647), Opa1 (CAS: sc-393296), Drp1 (CAS: sc-271583), and Fis1 (CAS: sc-98900) were obtained from Santa Cruz Biotechnology, Inc. (Santa Cruz, CA, USA).

### 2.2. Herbal Decoction Preparation

The preparation steps of QJJLD had been described in our previous study [10]. Briefly, all herbs were decocted for 45 min and filtered out the solution, then added 6-fold volume of water to redecoct for 30 min and filtered again. Finally, mixing these two times filtered solutions together in a water-bath at 75°C and letting it evaporate until attaining the concentration needed for the high-dose experimental group.

### 2.3. HPLC Analysis of QJJLD

The HPLC system was an Agilent 1260 Infinity HPLC system with a ChemStation for LC 3D systems (Rev.B.04.03-SP1, Agilent Technologies, USA). The compounds were chromatographically separated using a SB-C18 column (150 mm × 4.6 mm, 2.7 *μ*m, Agilent Poroshell, US) at 30°C. The mobile phase was a mixture of (A) 0.1% phosphoric acid-acetonitrile and (B) acetonitrile. The programmed gradient was as follows: 0 min, 15% B; 5 min, 15% B; 15 min, 35% B; 20 min, 45% B; 27 min, 52% B; 30 min, 65% B; 30.1 min, 95% B; 32 min, 95% B; 32.1 min, 15% B; 40 min, 15% B. The column was maintained at 30°C. The sample injection volume was 5 *μ*L. The wavelength of the ultraviolet (UV) detector was set at 230 nm (astragaloside IV, saikosaponin A, and saikosaponin D), 210 nm (calycosin-7-glucoside, liquiritin, hesperidin, lobetyolin, and atractylenolide III), 237 nm (monoammonium glycyrrhizinate), and 316 nm (ferulic acid, isoferulic acid) (40 min).

### 2.4. Experimental Animals and QJJLD-Containing Serum Preparation

Twenty-eight female Sprague-Dawley rats (6 weeks of age) were provided by Laboratory Animal Center of Guangzhou University of Chinese Medicine (Guangzhou, China). The experimental animal certification number was No. 44005800007597. The experimental scheme was authorized by the Institutional Animal Care and Use Committee of Guangzhou University of Chinese Medicine, and all the experiments followed the relevant regulations and guidelines. The rats were randomly divided into four groups: normal group, low-dose QJJLD group, middle-dose QJJLD group, and high-dose QJJLD group (Table S1). Rats in each group were given gavage for 7 days. There were a large number of researches [11–14] using drug-containing serum to study the mechanism of their protective effects. Based on the human equivalent dose in the clinical practice and the animals' surface area, we could covert human dose to the rat dose [15]. This scheme had the following properties: The low-dose QJJLD group was treated with QJJLD (5.85 g/kg·d). The middle-dose QJJLD group was treated with QJJLD (11.70 g/kg·d). The high-dose QJJLD group was given QJJLD (23.40 g/kg·d). Furthermore, all rats were given equal volume of liquid by gavage (10 mL/kg·d). One hour after the last treatment, the rats were anesthetized with pentobarbital sodium, and blood was collected from the abdominal aorta and allowed to stand at 37°C for 1 h. Serum was separated by centrifugation at 3000 rpm, filtered with a 0.22 *μ*m filter membrane, and stored at -80°C.

### 2.5. Cell Culture and Experimental Groups

Rat L6 myoblasts were purchased from the Cell Resource Center of Shanghai Institutes for Biological Sciences, Chinese Academy of Sciences (Shanghai, China). All cells were cultured in Dulbecco's modified Eagle's medium (DMEM) supplemented with 10% foetal bovine serum (FBS). The L6 myoblasts were seeded onto 60 mm culture dishes at a density of 2 × 10^5^ cells/mL. After culturing for 24 h, the cells were divided into six groups: normal group, model group, blank serum group, low-dose group, middle-dose group, and high-dose group. Except for the normal group, all groups were exposed to 0.8 mmol/L H_2_O_2_ for one hour. Afterwards, the normal group and model group were cultured in DMEM containing 10% FBS for 24 h, and the blank serum group was cultured in DMEM containing 10% blank serum for 24 h. Low-, middle-, and high-dose groups were cultured in DMEM with 10% low-, middle-, and high-dose QJJLD-containing serum, respectively, for 24 h.

### 2.6. Cell Viability Assay

Cell viability was detected by 3-(4,5-dimethylthiazol-2-yl)-2,5-diphenyltetrazolium bromide (MTT). The L6 myoblasts were seeded onto 96-well plates at a density of 1 × 10^5^ cells/mL. After corresponding treatment, 0.5 mg/mL of MTT was added to each pore and the cells were cultured for 3 h at 37°C. Then, the supernatant was interchange with the same amount of DMSO and dissolve the blue formazan crystals for 10 min. Finally, the optical density (OD) was detected at 490 nm with a VICTOR X5 Multilabel Plate Reader (PerkinElmer, USA).

### 2.7. Mitochondrial Ultrastructure

After corresponding treatment, the L6 cells were washed with PBS, fixed with 2.5% glutaraldehyde for 4 h, and then postfixed in 1% osmium tetraoxide for 2 h. Cells were dehydrated with concentration gradients of ethanol, embedded in Epon 812 resin, and then sectioned on an ultramicrotome (UC-7, Leica, Germany). Ultrathin sections were stained with 2% uranyl acetate and 2% lead citrate and then observed on a transmission electron microscope (JEM-1400, JEOL, Japan).

### 2.8. Detection of Reactive Oxygen Species

Rat L6 myoblasts were incubated with 10 mmol/L 2′,7′-dichlorofluorescein diacetate (DCFH-DA) at 37°C for 30 min. DCFH-DA was deacetylated by lactone and then oxidized by ROS to the fluorescence-causing substance, dichlorofluorescein (DCF). The fluorescence was then measured by fluorescence microscope (Olympus, Japan) and flow cytometry (Cytomics FC500, Beckman, USA). Fluorescence of 10,000 cells was measured and analyzed by the CXP Cytometer software to determine the intracellular ROS level.

### 2.9. Evaluation of SOD, GSH-Px Activity, and MDA Level

The corresponding kits (SOD, MDA, and GSH-Px) were provided by Nanjing Jiancheng Bioengineering Institute. All operations were performed according to the manufacturer's instructions.

Superoxide dismutase (SOD) activity in L6 myoblasts was determined by the WST-1 method. Briefly, cells were collected and sonicated after treatment. The supernatants were collected after centrifuging the cell lysate for 20 min at 3000 rpm. Protein concentrations were determined by a BCA Protein Assay Kit. Supernatants and double-distilled water were added into each well of a 96-well plate. The reaction solution was pipetted to each well and then incubated at 37°C for 20 min. SOD activities were subsequently measured by VICTOR X5 Multilabel Plate Reader (PerkinElmer, USA) at 450 nm and expressed as U/mgprot.

Malondialdehyde (MDA) activity in L6 myoblasts was determined by the TBA method. Briefly, cells were collected after treatment. The cell lysate by sonication was centrifuged at 3000 rpm for 20 min, and then, the supernatant was collected to measure the MDA levels. MDA can react with thiobarbituric acid (TBA) to form 3,5,5-trimethyloxazol-2,4-dione at high temperature. According to the extinction value at 532 nm, the MDA content can be calculated. The values were expressed as nmol/mgprot.

Glutathione peroxidase (GSH-Px) can catalyze the reaction of hydrogen peroxide (H_2_O_2_) with reduced glutathione (GSH) to produce H_2_O and oxidized glutathione (GSSG). So, the GSH-Px activity can be determined by measuring the consumption of GSH. GSH interacts with 5,50-dithiobis-2-nitrobenzoic acid (DTNB) to form 2-nitro-5- thiobenzoic acid which was quantified at 412 nm. According to the extinction value, the GSH content can be measured. GSH-Px activities were subsequently calculated and expressed as U/mgprot.

### 2.10. Mitochondrial Membrane Potential Evaluation

Rat L6 myoblasts were incubated in JC-1 staining working solution for 15 min at 37°C. MMP was then measured by fluorescence microscope (Olympus, Japan) and flow cytometry (Cytomics FC500, Beckman, USA). JC-1 aggregates in polarized mitochondrial matrix were detected in FL1 channel which emit red fluorescence at 590 nm when excited at 525 nm. JC-1 monomers in depolarized mitochondrial matrix were detected in FL2 channel which emit green fluorescence at 530 nm when excited at 490 nm.

### 2.11. Enzymatic Activity Assay of Mitochondrial Respiratory Chain Complexes I-IV

A spectrophotometric assay was performed according to the manufacturer's instructions to measure the enzymatic activities of respiratory complexes I-IV. Mitochondria were isolated from 5 × 10^6^ L6 myoblasts, further diluted in 202 *μ*L of assay buffer and sonicated. Complex I activity was evaluated by the oxidation rate of NADH at 340 nm. Complex II activity was determined by the reduction of dichlorophenolindophenol (DICIP) at 605 nm. Complex III activity was calculated by recording the increase in absorbance during the reduction of cytochrome C at 550 nm. Complex IV activity was measured by recording the decrease in absorbance during the oxidation of reduced cytochrome C at 550 nm.

### 2.12. Measurement of ATP Levels

The ATP content was measured using a commercially available ATP assay kit according to the manufacturer's instructions. Briefly, creatine kinase can catalyze the reaction of ATP and creatine to form phosphocreatine, and a colorimetric analysis of phosphomolybdic acid was used to determine the ATP level because the amount of phosphocreatine was proportional to the ATP content.

### 2.13. Quantitative Real-Time Polymerase Chain Reaction (PCR) Analysis

Total RNA from L6 myoblasts was extracted with TRNzol-A+ reagent. Then, the RNA was reverse transcribed into cDNA using a PrimeScript™ RT Master Mix Kit. Gene expression was analyzed by SYBR Premix Ex Taq™ II according to the manufacturer's instructions. Amplification was performed under the following cycling conditions on a 7500 Real-Time PCR System (Applied Biosystems, USA): 95°C for 30 s, followed by 40 cycles of 95°C for 5 s, 55°C for 30 s, and 72°C for 30 s. The primer sequences used for qRT-PCR are listed in Table 1. The relative gene expression level was calculated by the 2^-*ΔΔ*Ct^ method with GAPDH as a reference gene.

### 2.14. Western Blot Assay

L6 myoblasts were lysed in RIPA buffer and centrifuged at 12000 rpm. Protein concentrations were determined by a BCA Protein Assay Kit. Equal amounts of protein for each sample were separated by SDS-PAGE and transferred to PVDF membranes. The membranes were blocked with 5% nonfat dry milk for 1 h and subsequently probed with the primary antibodies for 12 h. Then, the membranes were incubated with secondary antibodies for 1 h. After visualized with enhanced chemiluminescence solution, the immunoreactive bands were visualized with an imaging station (ChemiDoc™XRS+, Bio-Rad, USA) and analyzed by the Image Lab software.

### 2.15. Statistical Analysis

All statistical data were analyzed by the SPSS 22.0 software (IBM, Armonk, NY, USA). The experimental data were presented as the mean ± standard deviation (SD). One-way ANOVA followed by Least-significant Difference (LSD) test was used for normally distributed data. Moreover, heteroscedasticity was analyzed by Dunnett T3 analysis. *p* < 0.05 or *p* < 0.01 was considered to indicate a statistical significance.

## 3. Results

### 3.1. HPLC Analysis

**Figure 1:**
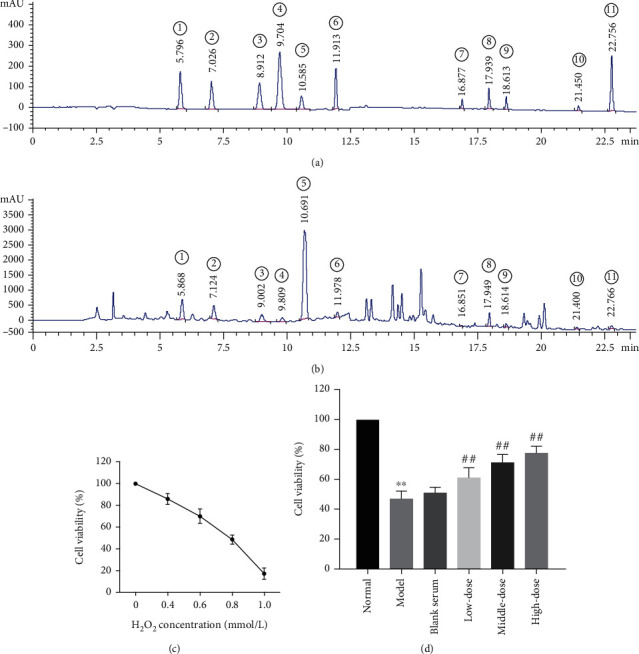
Effects of QJJLD-containing serum on the H_2_O_2_-induced oxidative stress damage. (a, b) HPLC chromatograms of the standard component mixture and QJJLD. ① Calycosin-7-glucoside, ② liquiritin, ③ ferulic acid, ④ isoferulic acid, ⑤ hesperidin, ⑥ lobetyolin, ⑦ astragaloside IV, ⑧ monoammonium glycyrrhizinate, ⑨ saikosaponin A, ⑩ saikosaponin D, and ⑪ atractylenolide III. (c) Cells were exposed to various concentrations of H_2_O_2_ for 1 h, and then, cell viability was assessed using the MTT assay (*n* = 3). (d) Attenuation of H_2_O_2_-induced cytotoxicity by QJJLD-containing serum in L6 myoblasts (*n* = 5). The results are shown as the mean ± SD values obtained from five independent experiments. Compared with the normal group, ^∗∗^*p* < 0.01 and ^∗^*p* < 0.05. Compared with the model group, ^##^*p* < 0.01 and ^#^*p* < 0.05.

The major components of QJJLD were analyzed by HPLC. By comparison with standard reference compounds, 11 compounds were identified in our study (Figures 1(a) and 1(b)). There are calycosin-7-glucoside, liquiritin, ferulic acid, isoferulic acid, hesperidin, lobetyolin, astragaloside IV, monoammonium glycyrrhizinate, saikosaponin A, saikosaponin D, and atractylenolide III.

### 3.2. QJJLD-Containing Serum Attenuated L6 Myoblasts against H_2_O_2_-Induced Cytotoxicity

As shown in Figure 1(c), the cell viability was significantly inhibited in a concentration-dependent manner, and the cells showed approximately 50% viability after exposure to 0.8 mmol/L H_2_O_2_. Therefore, the optimal concentration of H_2_O_2_ was selected as 0.8 mmol/L. To investigate the protective effect of QJJLD-containing serum on H_2_O_2_-induced cytotoxicity, L6 myoblasts were treated with various doses of QJJLD-containing serum for 24 h after exposed to 0.8 mmol/L H_2_O_2_ for 1 h. The results indicated that cell viability of the model group was reduced by approximately 50% (*p* < 0.01), and this reduction was dramatically protected by QJJLD-containing serum in a concentration-dependent manner (*p* < 0.01) (Figure 1(d)).

### 3.3. Effect of the QJJLD-Containing Serum on Mitochondrial Ultrastructure in L6 Myoblasts

**Figure 2:**
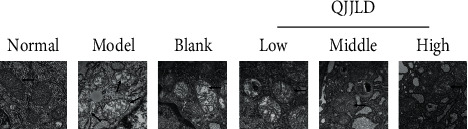
Representative TEM images of mitochondria. Normal mitochondria with intact outer membrane and dense cristae in normal group. Severely impaired mitochondria with fuzzy cristae and swelling matrix in model group and blank serum group. QJJLD-containing serum treatment improves mitochondria morphologies in low-, middle-, and high-dose groups (bar = 200 nm). The low-dose group has more mitochondrial cristae and little swelling matrix than model group. Moreover, the mitochondrial cristae and matrix in middle-dose group are better than that in low-dose group. The high-dose group also has more good contrast than middle-dose group.

Transmission electron microscopy was performed to observe mitochondrial ultrastructure. Mitochondria were observed with intact outer membrane and dense cristae in the normal group. H_2_O_2_ can provoke abnormal mitochondrial morphologies. The mitochondria were severely damaged with fuzzy cristae and swelling matrix in the model group. Some of them even appear disruptive membrane and disorganized vacuolization. QJJLD-containing serum treatment gradually attenuated mitochondrial damage, such as to improve swelling matrix, disarrayed cristae, and disruptive membrane which indicated that QJJLD plays a crucial role in maintaining normal mitochondrial ultrastructure (Figure 2).

### 3.4. Effect of the QJJLD-Containing Serum on ROS Generation in L6 Myoblasts

**Figure 3:**
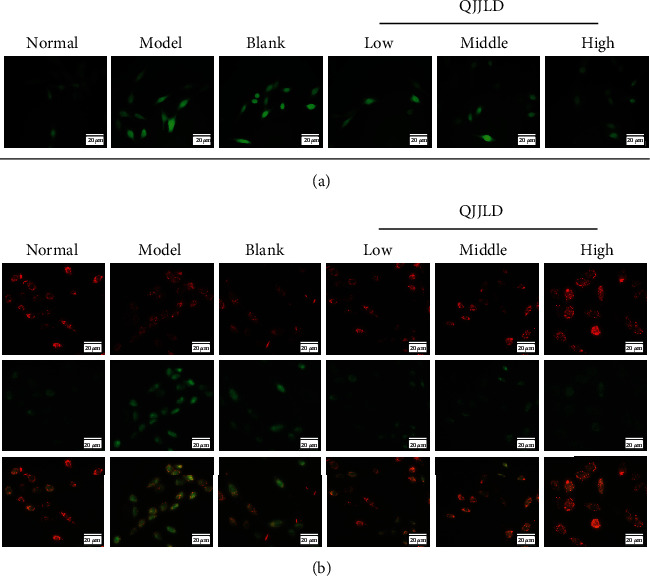
QJJLD-containing serum inhibited H_2_O_2_-induced increase of ROS level and restored the mitochondrial membrane potential in L6 myoblasts. (a) After treatment with 0.8 mmol/L H_2_O_2_ for 1 h, cells were incubated with or without QJJLD-containing serum for another 24 h. The decline in the membrane potential was reflected by the shift of fluorescence from red to green indicated by JC-1 (bar = 20 *μ*m). (b) Intracellular ROS level was measured by the fluorescence microscope (bar = 20 *μ*m).

**Figure 4:**
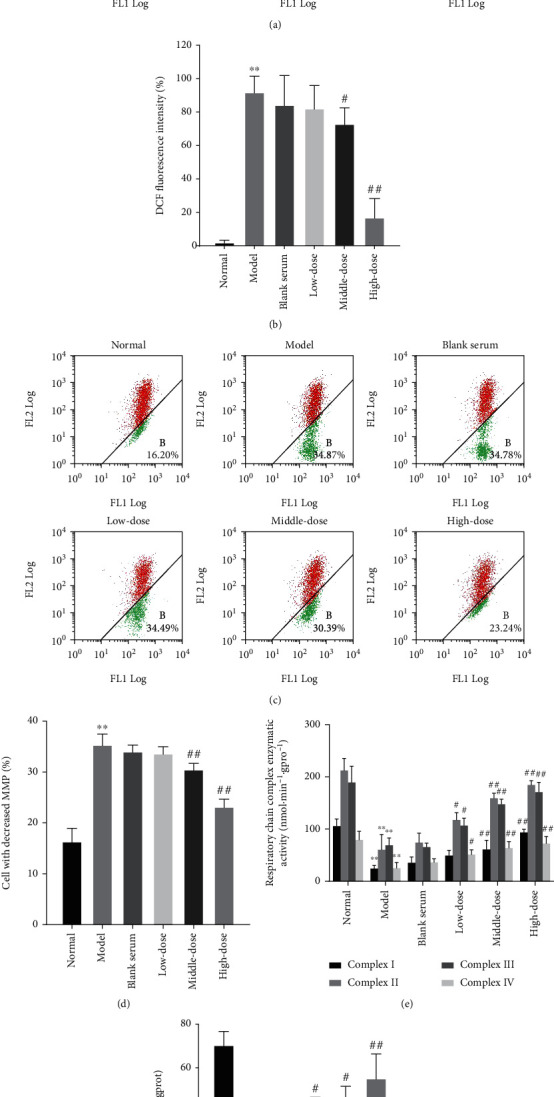
QJJLD-containing serum attenuated the H_2_O_2_-induced mitochondrial dysfunction in L6 myoblasts. (a, b) Effects of QJJLD-containing serum on intracellular ROS (*n* = 5). (c, d) Effects of QJJLD-containing serum on MMP in L6 myoblasts. The area of the A gate indicates the normal MMP, and the area of the B gate indicates the decreased MMP. The MMP change values were expressed as the ratio of A : B. (*n* = 5). (e) Effects of QJJLD-containing serum on the enzymatic activity of mitochondrial respiratory chain complexes in L6 myoblasts (*n* = 3). (f) Effects of QJJLD-containing serum on ATP content in L6 myoblasts (*n* = 3). The values are shown as the mean ± SD. Compared with the normal group, ^∗∗^*p* < 0.01 and ^∗^*p* < 0.05. Compared with the model group, ^##^*p* < 0.01 and ^#^*p* < 0.05.

The generation of ROS in the model group was markedly increased compared with that in the normal group after exposure to 0.8 mmol/L H_2_O_2_ for 1 h (*p* < 0.01). Exposure to QJJLD-containing serum exposure can reduce the oxidative stress level of the rat L6 myoblasts. After a 24 h incubation with QJJLD-containing serum, ROS generation was reduced in the middle (*p* < 0.05) and high-dose groups (*p* < 0.01) (Figures 3(a), 4(a), and 4(b)).

### 3.5. Effects of the QJJLD-Containing Serum on SOD, GSH-Px Activity, and MDA Level in L6 Myoblasts

**Figure 5:**
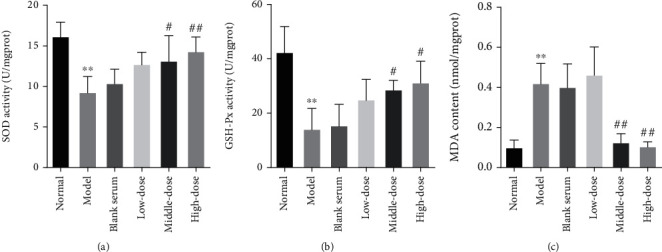
Effects of the QJJLD-containing serum on SOD, GSH-Px activity, and MDA level in L6 myoblasts. (a) SOD activity, (b) GSH-Px activity, and (c) MDA level. Data are shown as the mean ± SD of three separate experiments (*n* = 3). Compared with the normal group, ^∗∗^*p* < 0.01 and ^∗^*p* < 0.05. Compared with the model group, ^##^*p* < 0.01 and ^#^*p* < 0.05.

It has been demonstrated that SOD and GSH-Px protect the cells against H_2_O_2_-induced damages. To determine whether the protective effects of QJJLD-containing serum on L6 myoblasts are due to its antioxidant properties, SOD, GSH-Px activity, and MDA content were measured by commercial kits. As shown in Figure 5, MDA content was significantly elevated by 4.19 folds in cells exposed to H_2_O_2_ compared with the control group (*p* < 0.01). Interestingly, MDA content was dramatically decreased by treating L6 myoblasts with middle and high doses of QJJLD-containing serum (*p* < 0.01). In addition, the model group SOD and GSH-Px activities were obviously decreased by 1.74 and 2.99 folds, respectively. After treated with QJJLD-containing serum, SOD activity was elevated in middle-dose group (*p* < 0.05) and significantly increased in high-dose group (*p* < 0.01). GSH-Px activity was marked increased in middle- and high-dose groups (*p* < 0.05), compared with the model group.

### 3.6. Effect of the QJJLD-Containing Serum on MMP in L6 Myoblasts

The MMP was significantly decreased in L6 myoblasts in the group exposed to 0.8 mmol/L H_2_O_2_ for 1 h, compared with the normal group (*p* < 0.01). After treatment with different gradient doses of QJJLD-containing serum, the loss of MMP was significantly attenuated in the middle- and high-dose groups (*p* < 0.01) (Figures 3(b), 4(c), and 4(d)).

### 3.7. Effects of QJJLD-Containing Serum on the Enzymatic Activity of Mitochondrial Respiratory Chain Complexes

The enzymatic activity of mitochondrial respiratory chain complexes I-IV was significantly decreased in the model group (*p* < 0.01). After treatment with QJJLD-containing serum for 24 h, the activity of mitochondrial respiratory chain complexes I-IV in the middle- and high-dose groups was prominently increased compared with the model group (*p* < 0.01). The enzymatic activity of complexes II, III, and IV was increased in the low-dose group, compared with that in the model group (*p* < 0.05) (Figure 4(e)).

### 3.8. Effect of the QJJLD-Containing Serum on ATP Levels in L6 Myoblasts

The ATP contents of the model group were significantly reduced (*p* < 0.01). After QJJLD-containing serum treatment, ATP levels were increased in the low- and middle-dose groups (*p* < 0.05). ATP levels were also obviously raised in the high-dose group compared with the model group (*p* < 0.01) (Figure 4(f)).

### 3.9. Effect of the QJJLD-Containing Serum on p-AMPK, COX IV, PGC-1*α*, NRF1, and TFAM Protein Expressions in L6 Myoblasts

**Figure 6:**
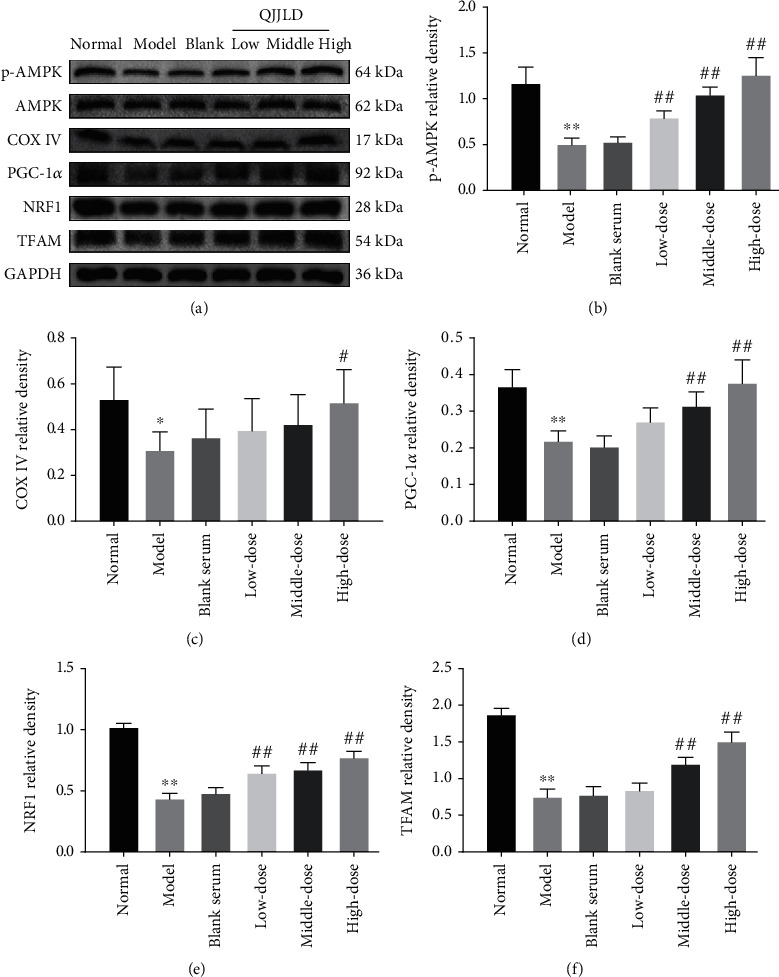
Effects of QJJLD-containing serum on the expression of proteins related to mitochondrial biogenesis. (a) The expression of phosphorylated AMPK, total AMPK, COX IV, PGC-1*α*, NRF1, TFAM, and GAPDH was detected by Western blot. (b) p-AMPK, (c) COX IV, (d) PGC-1ɑ, (e) NRF1, and (f) TFAM. Data are shown as the mean ± SD of five separate experiments (*n* = 5). Compared with the normal group, ^∗∗^*p* < 0.01 and ^∗^*p* < 0.05. Compared with the model group, ^##^*p* < 0.01 and ^#^*p* < 0.05.

The results show that the protein expression levels of p-AMPK, COX IV, PGC-1*α*, NRF1, and TFAM were significantly decreased in the model group compared with the normal group (*p* < 0.01). After QJJLD-containing serum treatment, the protein expression levels of p-AMPK, COX IV, PGC-1*α*, NRF1, and TFAM in the middle- and high-dose groups were significantly raised compared with the model group (*p* < 0.01). We also observed a marked upregulation of p-AMPK and NRF1 protein expressions in the low-dose group (*p* < 0.01). The protein expression of COX IV increased in the high-dose group (*p* < 0.01) (Figure 6).

### 3.10. Effect of the QJJLD-Containing Serum on Mfn1/2, Opa1, Drp1, and Fis1 mRNA Expressions in L6 Myoblasts

**Figure 7:**
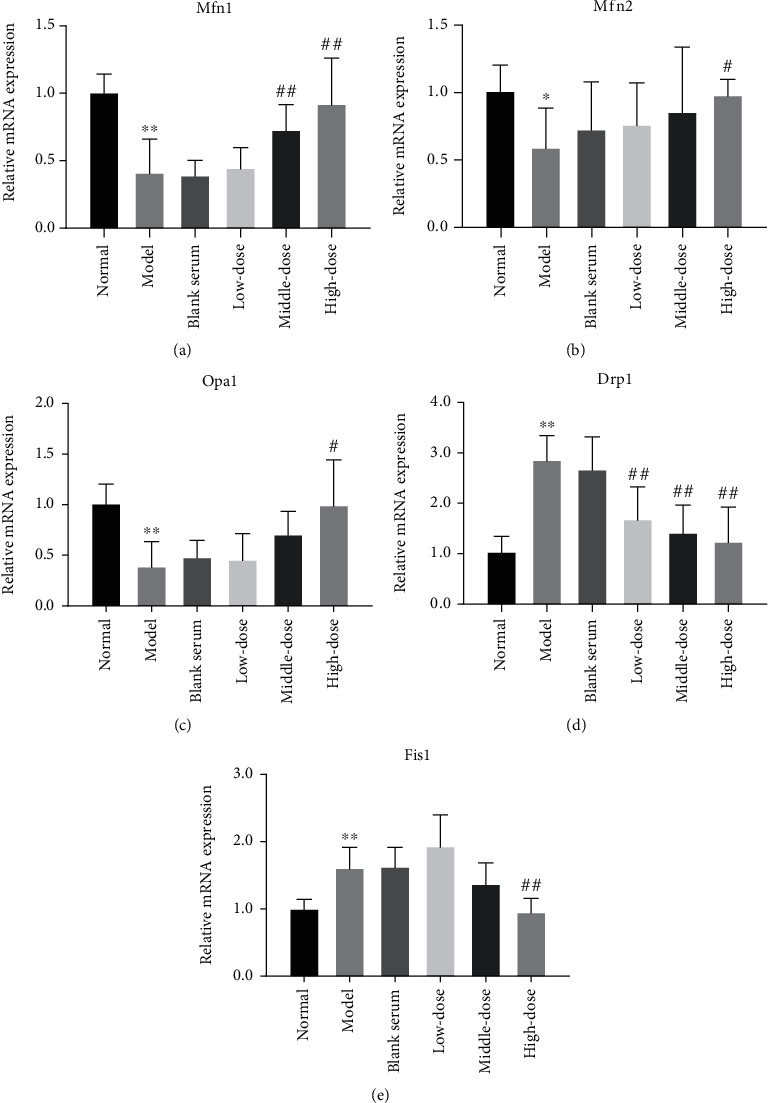
Effects of QJJLD-containing serum on mRNA expression related to mitochondrial fusion and fission. (a) Mfn1, (b) Mfn2, (c) Opa1, (d) Drp1, and (e) Fis1. The results are shown as the mean ± SD values obtained from five independent experiments (*n* = 5). Compared with the normal group, ^∗∗^*p* < 0.01 and ^∗^*p* < 0.05. Compared with the model group, ^##^*p* < 0.01 and ^#^*p* < 0.05.

mRNA expression of Mfn1/2 and Opa1 was decreased in the model group compared with the normal group (*p* < 0.01 and *p* < 0.05). After treatment with QJJLD-containing serum, Mfn2 and Opa1 mRNA expressions were raised in the high-dose group (*p* < 0.05). The Mfn1 mRNA expression level was significantly increased in middle- and high-dose groups (*p* < 0.01). In contrast, compared with the normal group, the model group had markedly increased Fis1 and Drp1 mRNA expression levels (*p* < 0.01). After treatment with QJJLD-containing serum, the mRNA expression level of Drp1 was inhibited in all the QJJLD-containing serum treatment groups (*p* < 0.01) while the Fis1 mRNA expression level was only decreased in the high-dose group (*p* < 0.01) (Figure 7).

### 3.11. Effect of the QJJLD-Containing Serum on Mfn1/2, Opa1, Drp1, and Fis1 Protein Expressions in L6 Myoblasts

**Figure 8:**
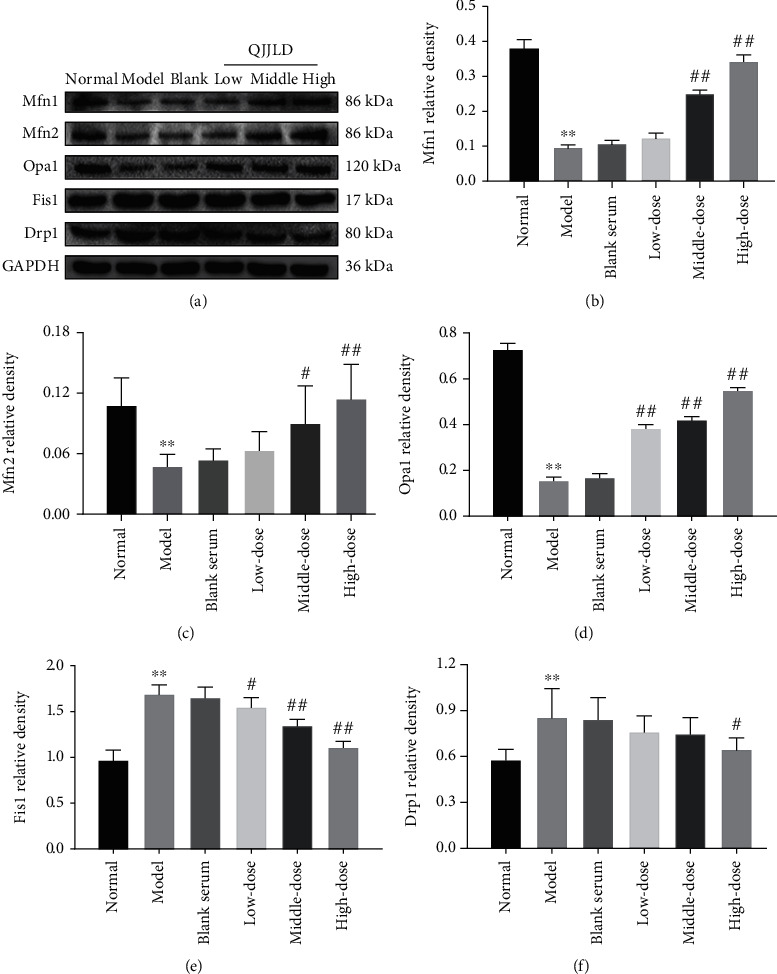
Effects of QJJLD-containing serum on the expression of proteins related to mitochondrial fusion and fission. (a) Western blotting was used to analyze the protein expressions. (b) Mfn1, (c) Mfn2, (d) Opa1, (e) Fis1, and (f) Drp1. Data are shown as the mean ± SD of five separate experiments (*n* = 5). Compared with the normal group, ^∗∗^*p* < 0.01 and ^∗^*p* < 0.05. Compared with the model group, ^##^*p* < 0.01 and ^#^*p* < 0.05.

Mitochondrial fusion is the repair system that corrects excessive mitochondrial fission by promoting mitochondrial communication [16]. The protein expression levels of Mfn1/2 and Opa1 were significantly lower in the model group than those in the normal group (*p* < 0.01). As shown in Figures 8(b)–8(d), the protein expression levels of Mfn1 and Mfn2 were increased in the middle- and high-dose groups compared with the model group (*p* < 0.05 and *p* < 0.01). Opa1 protein expression was significantly increased in all the QJJLD-containing serum treatment groups compared to that in the H_2_O_2_-treated group (*p* < 0.01). In contrast, the Fis1 protein expression level was decreased in all treatment groups compared with the model group (*p* < 0.05 and *p* < 0.01) while the Drp1 protein expression level was decreased in the high-dose group (*p* < 0.05) (Figures 8(e) and 8(f)).

## 4. Discussion

Accumulating evidence shows that the excessive ROS is directly related to muscle diseases associated with mitochondrial dysfunction [17, 18]. ROS are generated in various cellular compartments. However, mitochondria generate almost 90% of the cellular ROS. H_2_O_2_ can spread freely across the cell membrane and increase intracellular ROS especially in skeletal muscle [19]. In the current study, we evaluated the protective effects of QJJLD Cellular antioxidant systems, such as SOD and GSH-Px improve the ability of cells to alleviate mitochondrial damage caused by ROS [20]. Superoxide dismutase (SOD) is the primary enzymatic antioxidant to scavenge oxygen-free radicals including ROS. Moreover, the reaction of excessive ROS caused membrane lipid peroxidation and then compromised the membrane integrity. Moreover, MDA is an oxidative production of lipid peroxidation, which can affect the mitochondrial respiratory chain and the activity of key enzymes in mitochondria, thus leading to mitochondrial dysfunction [21] against H_2_O_2_-induced mitochondrial dysfunction and explored the underlying mechanisms. As the results show, cell viability was decreased in a concentration-dependent manner and was reduced by approximately half when L6 myoblast was incubated with 0.8 mmol/L H_2_O_2_ for 1 h (Figure 1(c)). After treatment with QJJLD-containing serum, the increased cell viability and the suppressed intracellular ROS generation indicated that QJJLD-containing serum can enhance muscle cell survival (Figures 1(d), 3(a), 4(a), and 4(b)). Then, we investigate the protective effect of QJJLD on the activity of antioxidant enzymes in L6 myoblast. The present study showed that MDA level was obviously increased after incubation with H_2_O_2_. We also observed a significant decrease of SOD and GSH-Px activities revealing an insufficient competence of the antioxidant system to detoxify ROS in L6 myoblasts. QJJLD-containing serum treatment resulted in an increase of SOD and GSH-Px activities which subsequently contributed to the ROS scavenging and MDA reduction in L6 myoblasts (Figure 5). These results indicated that QJJLD could effectively alleviate the H_2_O_2_-induced injury on antioxidant system in L6 myoblasts.

The respiratory chain located in the inner mitochondrial membrane and its oxidative phosphorylation (OXPHOS) system is composed of complexes I-V. Complex IV is proposed to be involved in regulation of the respiratory rate and proton translocation; thus, this complex has a profound influence on OXPHOS [22]. Complexes I and III can associate with complex IV to form supercomplexes, which may not only modulate ROS formation but also establish the electrochemical proton gradient for complex V to synthesize ATP [23]. Impaired electron transfer in mitochondria respiratory chain can induce an increase in ROS generation and decrease in ATP production [24, 25]. After treatments, the activity of mitochondrial respiratory chain complexes I-IV of L6 myoblasts was obviously upregulated. We then measured the ROS level and ATP content in L6 myoblasts. As shown in Figures 3(a), 4(a), and 4(b), the ROS level in the treatment groups was less than that in the model group while the ATP content was higher in the treatment groups. Our results revealed that QJJLD-containing serum can inhibit the ROS generation, alleviate mitochondrial respiratory chain damage, and promote mitochondrial energy metabolism in L6 myoblasts.

The energy which released from the electron transport chain is used to pump protons out through the inner mitochondrial membrane, finally forming an electrochemical gradient to maintain the resting membrane potential by active ion pumping [26]. The MMP is normally maintained at −120 to −180 mV, but inhibition of the mitochondrial complexes will lead to mitochondrial depolarization which is characterized by MMP collapse [27]. MMP can reflect the mitochondrial function because increased MMP will lead to ATP generation and increase mitochondrial oxygen consumption [28]. This is consistent with the results of our experiment. Our data showed that both MMP and ATP levels increased after treatment with QJJLD-containing serum. These investigations indicated that QJJLD-containing serum could alleviate MMP collapse and promote mitochondrial energy metabolism.

Mitochondrial biogenesis can increase the capacity of oxidative phosphorylation and is critical for skeletal muscle function. PGC-1*α* is a key regulator of mitochondrial biogenesis [29]. NRF1 can act on several genes required for mitochondrial respiratory function and modulate energy supply [30, 31]. TFAM is also a key molecule in regulation of mtDNA copy number [32]. PGC-1*α* activates NRF1 which can further the activity of TFAM, thus initiating the mitochondrial biogenesis and increasing intracellular ATP levels. The dysregulation of mitochondrial biogenesis results in ATP depletion and excessive ROS generation [33]. A previous study showed that AMPK activation can promote the transcription of PGC-1*α*, and AMPK can directly phosphorylate PGC-1*α* to increase its activity [34]. Both activation of AMPK and PGC-1*α* can further increase the expression of cytochrome c oxidase (COX IV) to enhance the activities of mitochondrial respiratory chain. In patients with statin-induced myopathy or in the skeletal muscle of rats treated with high doses of statins, ROS production was much higher, which subsequently suppressed mitochondrial biogenesis and resulted in mitochondrial dysfunction [35]. Metformin, through activating PGC-1*α* via AMPK induction, has recently been shown to improve the mitochondrial network and inhibit ROS generation to attenuate mitochondrial dysfunction in heart fibroblasts [34]. Astragaloside IV, one of the QJJLD effective compounds, can promote the phosphorylation of AMPK and activate PGC-1*α* to enhance energy metabolism and inhibit skeletal muscle cell apoptosis [36]. Besides, human fibroblast growth factor 19 (FGF19) can promote mitochondrial biogenesis and the antioxidant response through the AMPK/PGC-1a pathway and alleviate mitochondrial dysfunction [37]. In the AMPK-deficient HUVECs, PGC-1*α* mRNA and protein levels were significantly reduced, suggesting that AMPK is an upstream regulator [38]. AMPK was downregulated by Compound C (AMPK inhibitor); the mRNA and protein expression levels of its downstream effectors were significant decrease, including PGC-1*α*, NRF1, and TFAM [37]. Knockout of AMPK was also shown to reduce the expression of various genes related to mitochondrial biogenesis in mouse skeletal muscle [39]. Our results demonstrated that the p-AMPK, PGC-1*α*, NRF1, TFAM, and COX IV protein expressions were increased in all treatment groups compared with the model group (Figure 6). Combined with the decreased ROS level and increased ATP content in the treatment groups, we proposed that QJJLD-containing serum exerted its protective effects via enhancing mitochondrial biogenesis to prevent H_2_O_2_-induced mitochondrial dysfunction.

**Figure 9:**
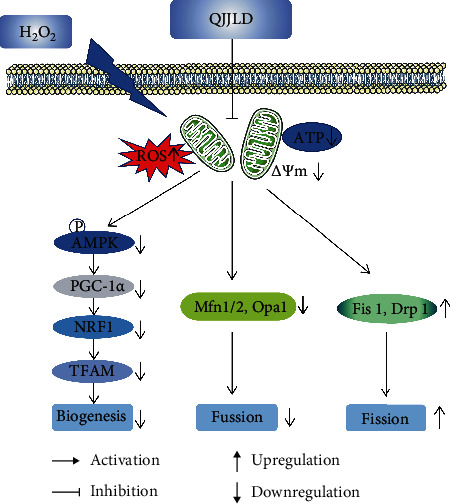
The possible mechanism of QJJLD. Excessive H_2_O_2_ caused the cellular ROS level increased, and MMP and ATP were reduced in L6 myoblasts. Administration of QJJLD significantly inhibited H_2_O_2_-induced imbalance of biogenesis and mitochondrial dynamics. QJJLD may alleviate mitochondrial dysfunction through the regulation of mitochondrial dynamics and biogenesis, the inhibition of ROS generation, and the promotion of mitochondrial energy metabolism. Therefore, QJJLD may have a beneficial effect on the therapy of muscle diseases associated with mitochondrial dysfunction.

**Figure 10:**
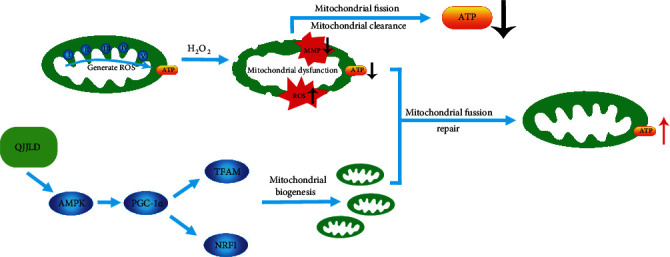
The protective effects of QJJLD on mitochondria. H_2_O_2_ induced mitochondrial respiratory chain damage, increased the cellular ROS level and consequently reduced ATP content. QJJLD could up-regulate the p-AMPK, PGC-1*α*, NRF1, and TFAM to promote mitochondrial biogenesis, which resulted in increasing mitochondria number by generating more new mitochondria. The mitochondrial fusion could help the new-generated-functional mitochondria supplement the old-mild-impairment mitochondria through the diffusion between organelles and the sharing of components. The mitochondrial fission would clear the old-severe-impairment mitochondria which resulted in the reduction of ATP. The upregulated mitochondrial fusion and downregulated fission gradually improved the mitochondrial morphology, such as to improve swelling matrix, disarrayed cristae, and disruptive membrane.

Mitochondria are highly dynamic organelles that are constantly remodeled by fusion and fission. Mitochondrial dynamics can maintain mitochondrial morphology adapted for maximal ATP production. Mfn1 and Mfn2 are important for outer membrane fusion, whereas Opa1 is necessary for inner membrane fusion. Mitochondrial fusion prevents cellular damage by allowing functional mitochondria to supplement dysfunctional mitochondria through the diffusion between organelles and the sharing of components [40]. This process can maintain ATP production and promote cell survival [28]. Fis1 helps Drp1 assembles to the outer mitochondrial membrane and modulates mitochondrial fission. The overexpression of Drp1 or Fis1 disrupts mitochondrial reticulation resulting in the fragmentation of mitochondria, loss of MMP, suppression of electron transfer chain activities, and mitochondrial metabolism, thus finally facilitating apoptotic cell death [41]. Notably, QJJLD-containing serum decreased the protein expression of Fis1 and Drp1 to inhibit mitochondrial fission. In addition, QJJLD markedly increased the mRNA and protein expressions of Mfn1/2 and Opa1 to promote mitochondrial fusion (Figures 7 and 8). In accordance with previous experiments, as mentioned above, our results show that the enzymatic activity of respiratory chain complexes I-IV, intracellular ATP content, and MMP were increased after treatment. Moreover, our results show that QJJLD-containing serum treatment gradually attenuated mitochondrial damage, such as to improve swelling matrix, disarrayed cristae, and disruptive membrane (Figure 2). These observations indicate that QJJLD-containing serum could also regulate mitochondrial dynamics to repair mitochondrial dysfunction and promote mitochondrial energy metabolism. To pose a hypothesis to explain the observations of our results, we suggest that QJJLD can upregulate the p-AMPK, PGC-1*α*, NRF1, and TFAM to promote mitochondrial biogenesis. As mitochondrial biogenesis can increase mtDNA copy number and protein synthesis, it is reasonable that mitochondrial respiratory chain damage is relieved by upregulation of complex I-V protein synthesis. The improvement of mitochondrial respiratory chain goes on to increase ATP production and decrease ROS generation. Mitochondrial biogenesis also can result in increasing mitochondria number by generating more new mitochondria and thus to produce more ATP. Enough ATP can support mitochondrial fusion to help new-generated-functional mitochondria supplement old-mild-impairment mitochondria through the diffusion between organelles and the sharing of components. The mitochondrial fission will clear old-severe-impairment mitochondria which is difficult to repair. The upregulated mitochondrial fusion and downregulated fission gradually improve mitochondrial morphology, such as to improve swelling matrix, disarrayed cristae, and disruptive membrane. Finally, QJJLD protects against H_2_O_2_-induced mitochondrial dysfunction by regulating mitochondrial dynamics and biogenesis (Figures 9 and 10).

## 5. Conclusions

The present study demonstrated that mitochondrial dysfunction is closely associated with H_2_O_2_-induced ROS generation. The results presented in this study provided the first evidence that QJJL decoction could act on skeletal muscle cells to ameliorate mitochondrial dysfunction by decreasing ROS level, increasing MMP, improving mitochondrial ultrastructure, and promoting intracellular ATP production. Simultaneously, the protein expressions of p-AMPK, PGC-1*α*, NRF1, and TFAM were upregulated. The mRNA and protein expressions of Mfn1/2 and Opa1 were also upregulated while Drp1 and Fis1 were downregulated. On the basis of our results, we suggested that QJJLD might regulate mitochondrial dynamics and biogenesis in L6 myoblasts to protect against H_2_O_2_-induced mitochondrial dysfunction. These findings suggested a therapeutic strategy for mitochondrial dysfunction-related diseases.

## Figures and Tables

**Table 1:** Primer sequences used in the qRT-PCR assay.

Gene	Forward	Reverse
Mfn1	5′-AGATAATGCAGCCCAGGAAGAG-3′	5′-GCACGAGTAGTCCAAGTCAGT-3′
Mfn2	5′-GAGTGTCAAGACCGTGAACCA-3′	5′-CATCCAGGCAAAACTTATCAATCCA-3′
Opa1	5′-TGGGAAGGGCTTTTCTGATTT-3′	5′-TCTGCTGTTGGAGGTGGCTAT-3′
Drp1	5′-ATTCTTCGGTTCATCAGTAATCCCA-3′	5′-AATAACCCTTCCCATCAATACATCC-3′
Fis1	5′-TGAATACGCCTGGTGCCTGGTT-3′	5′-TCCCGCTGCTCCTCTTTGCTAC-3′

## Data Availability

The data used to support the findings of this study are available from the corresponding author upon request.

## Supplementary Materials

Supplementary Materials Table S1: SD rats for QJJLD-containing serum preparation.
