# Supplementary material for: Qiangji Jianli Decoction Alleviates Hydrogen Peroxide-Induced Mitochondrial Dysfunction via Regulating Mitochondrial Dynamics and Biogenesis in L6 Myoblasts
Source: Oxid Med Cell Longev. 2021 Apr 13;2021:6660616. doi: 10.1155/2021/6660616 (PMC8060107; doi:10.1155/2021/6660616)
Supplement: Supplementary Materials — Table S1: SD rats for QJJLD-containing serum preparation. [file 6660616.f1.pdf]

Table S1: SD rats for QJJLD-containing serum preparation.

Group	number	average weight (g)	Treatment
normal group	7	236.04±5.57	equal volume of liquid
Low-dose group	7	234.69±4.08	5.85g/kg·d QJJLD
Middle-dose group	7	234.73±6.70	11.70 g/kg·d QJJLD
High-dose group	7	236.63±5.64	23.40g/kg·d QJJLD

Data were expressed as Mean ± SD (n=7). Before QJJLD-containing serum preparation, the average weight of 28 SD rats is 235.52±5.32g, and there is no statistical significance of the average weight among the four groups.
